# Personal responsibility for health in Bulgarian public health law and social legislation

**DOI:** 10.1186/s12913-024-11624-6

**Published:** 2024-10-05

**Authors:** Silviya Aleksandrova-Yankulovska, Florian Steger

**Affiliations:** Institute of the History, Philosophy and Ethics of Medicine, Ulm University, Ulm, Germany

**Keywords:** Bulgaria, Public health law, Personal responsibility for health, Prospective responsibility, Resource allocation, Retrospective responsibility, Social legislation

## Abstract

**Background:**

In the last decades all health systems have experienced a lack of resources. Against this background, the idea of ​​applying personal responsibility of the patient as a criterion for allocation of resources (PRCAR) is gaining increasing attention. Bulgarian healthcare reform has been marked by the implementation of many new strategies, that grounded our scientific interest towards investigating PRCAR in Bulgarian public health law and social legislation.

**Methods:**

Through a search of national legal databases 7 documents were selected and subjected to content analysis.

**Results:**

Prospective responsibility was found in two and retrospective responsibility - in three documents, two of which imposed explicit penalties on the patient. Two documents did not distinguish between the types of patient responsibility. PRCAR was found to be controversial through the prism of the social justice principle. The discussion was conducted through the perspectives of evidence translation of research to law, particularities of social cohesion in Bulgaria, and the interpretation of principles of public health ethics.

**Conclusion:**

Although PRCAR was traceable in Bulgarian legislation, no supporting arguments for its introduction were deduced. The applicability of PRCAR should be further studied and wider public debate should be initiated.

## Key points

• In the context of insufficient healthcare resources experienced by all healthcare systems, the idea of ​​applying the personal responsibility of the patient as a criterion for the allocation of resources (PRCAR) is appealing.

• If striving for a common European vision on PRCAR, then each European country’s view on the subject would be of key importance, and studies in this direction are necessary.

• Our manuscript offers an in-depth expert analysis of the presence of PRCAR in public health law and social regulation in Bulgaria, which is one of the more recent EU member states but experiences the same developmental trends as other European healthcare systems.

• As PRCAR is still an under-researched concept, our results will be of particular interest to other public health researchers.

## Introduction

In the second half of the 20th century all healthcare systems began to experience a lack of resources against the background of a continuous increase in the consumption of healthcare. Resource allocation has become not only an important healthcare management problem but also a priority topic in the field of public health ethics. In the context of the concept of health promotion, which has developed intensively since the 80s of the 20th century, the idea of ​​applying the personal responsibility of the patient as a criterion for the allocation of resources (PRCAR) in health care is gaining more and more supporters.

Two concepts of personal responsibility have been differentiated. Much of the bioethics literature has been focused on retrospective responsibility, i.e. whether people should receive lower priority access to health care if they are responsible for their illness. The prospective responsibility has gained more attention just recently. It is focused on preventive, responsible behaviour to avoid future negative consequences for the health of the individual, third parties, and society as a whole [1]. Within this concept, new variations have arisen that spark ethical debates. Such is Feiring’s model [2] according to which an individual who has causally contributed to their condition should be given equal priority only at the first instance of medical need but further needs to commit to a lifestyle contract, i.e. engagement with certain health behavioural changes. Disagreement or non-compliance with the lifestyle contract would result in a lower priority of access to public health care. Feng-Gu proposed a modified Feiring model [3] which places more emphasis on the nature of the lifestyle change attempt rather than on the result.

Against the background of the still ongoing debate on retrospective versus prospective responsibility in health policies for resource allocation [4], the first examples of lifestyle-dependent access to health care already exist [5]. One in three clinical commissioning groups in England refuse or delay elective operations on patients such as hip and knee replacements until patients stop smoking or lose weight. This applies to non-urgent elective surgical procedures [6], with various exclusion criteria [7].

In Germany, the 2007 legislative reform took a step toward greater consideration of personal responsibility with the introduction of additional patients’ contributions in non-medically indicated procedures, incentives for participation in preventive measures, and chronically ill patients’ compliance [8]. In general, however, the health insurance law in Germany has so far refrained from assigning central importance to the idea of personal responsibility.

To date, large-scale surveys of the medical community’s or general public’s opinion on PRCAR have not been conducted. However, the results of several small national studies are similar. 65.4% of participants in a British survey [3] supported limited access to health care in cases of repeated breaches of the lifestyle contract. A large-scale survey by Everett et al. [4] among 428 medical professionals in Great Britain and 1141 doctors in Norway showed no definite preponderance of opinion, except the support (57.98% and 67.43%, respectively) for additional payments on patients responsible for their illness. This was supported by only 11% of Swedish doctors [9], but 83.9% supported smoking cessation as a required lifestyle change before hip replacement in a consequent study [10]. Traina [11] found controversial opinions among Norwegian clinicians about the introduction of a formal prioritisation criterion based on individual responsibility.

Since 1990, Bulgaria has undergone a period of profound healthcare reform to improve the effectiveness of the healthcare system. The reform has affected in parallel the structure of the healthcare system with the introduction of new types of healthcare institutions and the type of financing from a state monopoly system to a social health insurance model. Corresponding healthcare legislation was adopted and many modern approaches to disease prevention and health promotion were introduced [12]. Within the first priority area of the National health strategy 2021–2030 is health promotion [13]. Its implementation opens opportunities for new approaches. All of this has grounded our scientific interest towards investigating the novel concept of personal responsibility in Bulgarian public health law and social legislation.

## Methods

Our research question is: whether the concept of personal responsibility is present in Bulgarian public health law and social legislation.

The search for relevant documents was done in two phases. First, legal documents were derived from the Bulgarian national legal portal: Lex.bg. These included the three main laws regulating the Bulgarian healthcare system, i.e. Bulgarian Health Act, the Law on Health Insurance, the Law on Health Institutions, and the main law on social security, i.e. Social Security Code. The Code of Professional Ethics of Physicians in Bulgaria was also in the first line of selected documents. Legal documents of the lower hierarchy, that were referred to in the main laws, were also subjected to keywords-driven search. The core set of key words included: “responsibility”, “personal responsibility”, and “patient’s responsibility”. We searched for these keywords in the texts of the documents. Second, we searched national health strategies. The respective keywords searched in the titles and within the texts included: “health strategy”, “smoking”, and “obesity”.

The study presents documentary research based on national legal documents and strategies. Only documents that are currently in force were included. Documents that did not contain provisions on personal responsibility for health were excluded. Altogether 7 documents were selected and subjected to content analysis. The content of the documents was analysed manually and systematically categorised based on the following criteria: issuing institution, a corresponding type of personal responsibility, and the resulting penalty on the patient. National sociological studies and official institutional reports were also reviewed.

## Results

We have identified 7 documents that contain texts relevant to the research question (Table 1).

**Table 1:** Outline of the national documents included in the analysis

Document	Issuing institution	Type of personal responsibility	Resulting penalty
Bulgarian Health Act	Bulgarian Parliament	Prospective responsibility	Implicit
Law on Health Insurance	Bulgarian Parliament	Retrospective responsibility	Explicit
Social Security Code	Bulgarian Parliament	Retrospective responsibility	Explicit
Code of professional ethics of physicians in Bulgaria	Minister of Health after adoption of Bulgarian Medical Association	Prospective responsibility	Implicit
Regulation no. 17 of May 27, 2004 on the conditions and procedure for inclusion of persons in need of organ transplantation in the official register of the executive agency “Medical supervision” and for the selection of a specific recipient of an organ, tissue or cells	Minister of Health	Retrospective responsibility	Implicit
National Health Strategy 2021–2030	Bulgarian Parliament	Not distinguished	No
Recommendations for good clinical practice in obesity	Minister of Health	Not distinguished	No

### Main laws

In the order of hierarchy, we started with the Bulgarian Health Act. In the background of the constitutionally guaranteed right to health care, no explicit texts referred to the concept of personal responsibility. However, personal responsibility of the prospective type was implied in Art.94, stating the duty of the patient to take care of his own health, among other patient’s duties [14].

Next, direct personal retrospective responsibility was implied in the Law on Health Insurance, Art. 111, where persons who have damaged their own health or have damaged the health of others in a state of alcoholic intoxication or drug abuse, must reimburse the healthcare costs to the National Health Insurance Fund. For the due amount of money, the Regional Health Insurance Fund may request the issuance of an order for immediate execution in accordance with Art. 418 of the Civil Procedure Code [15]. Thus, the resulting penalty for the patient is imminent and explicit.

Clearly defined retrospective personal responsibility was found in the Social Security Code, Art. 46, according to which monetary compensation for temporary incapacity is not paid to insured persons who intentionally damage their health to receive leave or compensation, violate the regime determined by the health authorities, have become unable to work due to the use of alcohol or narcotic agent, have become unable to work due to hooliganism and other anti-social behaviour, or have become unable to work due to non-compliance with the rules for safe work [16]. The negative consequences on the patient are explicit.

### Other studied documents

Of particular interest for our study was the Code of Professional Ethics of Physicians in Bulgaria as a document that provides the basis for professional norms of conduct. A broader interpretation of Art.12 of the Code correlated with the idea of prospective responsibility. Apparently, the physician has the right to refuse medical care on the grounds of lack of trust except in emergencies [17]. Patient’s non-compliance with prescriptions, on the other side, is a cornerstone reason for distrust in the therapeutic relation and it was among the key criteria for imposing prospective responsibility in the referred studies in Norway and the United Kingdom [4].

Of the reviewed ordinances, we have found an implicit criterion of the patient’s responsibility in the liver transplant algorithm of the Recipient Selection Ordinance. Art. 2 stipulates: *“*The purpose of this regulation is to ensure equal access of citizens to transplantation based entirely on medical selection criteria”. However, the described procedure of recipients’ assessment, includes accumulation of points. The detailed instructions distribute the points as follows: “need for urgent retransplantation (failure of the transplanted liver in the first week after transplantation) = 30 points; fulminant liver failure in the absence of previous liver disease = 20 points; other reason = 0 pts.” [18]. Therefore, if the patient needs liver transplantation due to alcoholic cirrhosis, he would not gain any points, which dooms him to remain at the end of the waiting list.

Lastly, we have looked into the national health strategies targeting smoking and obesity, the two criteria that have been already applied in some international prioritisation policies [5].

The Bulgarian National Health Strategy 2021–2030 is an official document adopted by the Parliament and the activities in line with it are funded by the state budget. Smoking is targeted in Priority 1: Public health - strengthening the capacity of public health; Health promotion and disease prevention [13]. A variety of health promotion activities are envisaged to increase public awareness of the effects of the risk factors, but no notion of personal responsibility could be traced in the document. The susceptible groups were guaranteed equal access to services and activities related to health promotion and reduction of risk factors.

Similarly, in the “Recommendations for good clinical practice in obesity” [19] different approaches to achieve behavioural change and reduction of Body-Mass-Index are described. However, it is done more in the form of instructions to physicians and general recommendations. Thus, they miss the active involvement of the patient and any implication for the patient’s responsibility. They are also in no way related to limited access to further health care.

## Discussion

Regardless of the high hopes placed on the concept of health promotion to save healthcare resources, the health systems kept being overwhelmed by unmet patient needs and growing expenses [20] which in turn necessitated the search for new strategies and partnerships. The latter presumes a change in the role of the patients from consumers of health services to well-informed individuals who are enabled to make responsible decisions for their health in the context of favourable conditions provided by the states and communities. In such a scenario it seems justified to hold patients responsible for their lifestyle choices and related health status. However, this contradicts the social justice principle, that has been long proclaimed in contemporary medicine and medical ethics. The Declaration of the World Medical Association (WMA) on the rights of patients states that any choice between patients for treatment should be based on medical criteria only [21]. Thus, a choice based on patients’ lifestyle is precluded because it cannot be considered a “medical criterion” and would easily qualify as discrimination.

### PRCAR in the studied documents

Despite this controversial ethical background, the concept of patient responsibility is traceable in Bulgarian health and social legislation. While in the Bulgarian Health Act and the Code of Professional Ethics of Physicians in Bulgaria it is very subtle and leaves much room for interpretation, the other laws are more specific and bind the concept with concrete negative consequences for the patient. Still, these do not go so far as to be associated with the notion of the lifestyle contract. The provisions in the Law on Health Insurance and the Social Security Code seem to be driven more out of concern to prevent abuse of public funds than out of health promotive goals. From a consequential point of view, however, they reach the same effect, namely to warn people against intentional damage to their health. There is no room for deeper interpretations and it certainly does not extend to the notion of the lifestyle contract and does not distinguish prospective and retrospective types of personal responsibility.

One of the most controversial positions that we found is the one in the Code of Professional Ethics of Physicians in Bulgaria, as it goes against the traditional unconditional dedication of the physician to the patient. However, in line with the growing importance of respecting the autonomy of both parties entering into a therapeutic relationship, it is understandable to also encounter provisions for respecting the physician’s autonomy, as long as they are balanced with the right of the patient to medical care. In our case, this is achieved in Art.24 through the unconditional duty to provide emergency care and the duty to refer the patient to another colleague in the event of the physician’s withdrawal [17].

As for the algorithm for the choice of recipient of a liver transplant, it does raise ethical concerns. On the one hand, personal responsibility is only implicit and there is no clear mention of a deleterious lifestyle. Thus, it could be argued that this is just an overly broad interpretation on our part. On the other hand, alcoholics will inevitably be non-prioritised according to the scoring procedure described. Moreover, none of the other algorithms in the same ordinance, i.e. for kidney, heart, and lung transplantation, contain a similar point system. We then have at hand an objective conclusion of imposed personal responsibility that contradicts the ethical guidelines in place. The WMA Statement on organ and tissue donation clearly proclaims that the choice between recipients should be based on “severity and urgency of medical need, length of time on the waiting list and medical probability of success”. Social status, lifestyle, and behaviour should not be used as allocation criteria [22]. On the other hand, the generally deteriorated health status of these patients already puts them towards the end of the waiting list because of the “low medical probability of success”. Thus, we can argue that some “objective” criteria for resource allocation are already distorted and discriminatory because of the interaction of different lifestyle factors.

### Introduction of new legal regulation

Law is often used as a tool of intervention. However, the successful development and implementation of the law depends on a well-cultivated partnership with all stakeholders [23]. Such partnership extends to all activities accompanying the establishment of the law: (1) research on most strategically relevant public health questions [24]; (2) development of model laws based on the best available science [25]; (3) complex community education and advocacy campaigns; and (4) monitoring public health outcomes [23]. Thus, it is critical to first conduct research on important public health issues, then the translation of this research experience into effective legislation, and lastly, monitoring and evaluation of legislation’s impact. From this perspective, we can question the evidence on which the laws, adopting PRCAR, step. At least for the Bulgarian national context such research data are absent. As for the international context, we have shown that the available research is rather narrow, small-scale, and nationally focused, which makes the data not representative. The successful implementation of public health laws and the attainment of their goals require active collaboration on the side of the targeted communities. These should be reached through health education strategies and advocacy campaigns. In the Bulgarian context, though, such campaigns have not taken place, which additionally undermines the public support of the concept of patient responsibility. It is even more valid for Bulgarian society, in which social cohesion is estimated to be low [26]. Social cohesion is “the property by which the whole society, and individuals within, are bound together through the action of specific attitudes, behaviours, rules, and institutions, which rely on consensus rather than pure coercion” [27]. Among the key measurable features of social cohesion is the level of trust [28] which has been assessed as low in Bulgarian society [29]. Societies with higher levels of social cohesion have been shown to be generally healthier [30]. One striking example of the effect of reduced social cohesion in Bulgaria was revealed during the COVID-19 pandemic, where Bulgaria registered the lowest rates of uptake of at least one dose of vaccine among the EU member states, namely 30.5% versus 82.1% in Ireland and 77.9% in Germany [31]. All of this happened in the background of mistrust in the offered prevention measures. The interests of public health and the safety of other members of society took a back seat. These lessons from history, together with the consideration of national particularities, must be considered when introducing new public health policies.

About the monitoring of public health outcomes, we looked at the official reports of impact assessment of the investigated legal documents. Impact assessment is a formal mechanism that systematically examines and analyzes the impact is of planned and implemented public policies according to a strictly defined algorithm [32]. As for the available impact assessments of the documents that we studied, we found that they do not include any special consideration of the texts related to the individual responsibility of patients.

### Principles of public health ethics applied

Further, we have examined the Bulgarian case of the legal implementation of PRCAR through the prism of the principles of public health ethics, namely the principles of imposing harm on the separate individual for the benefit of the society (harm principle); effectiveness; proportionality; necessity; least infringement; public justification; and reciprocity [33, 34].

The application of the harm principle would lead to individual patients being denied access to certain healthcare measures based on their responsibility for their health status. This would be justifiable as long as there is a clear benefit for the society. Such is presumed with the saved resources from the particular patient and their redistribution to the other patients in need. However, such an effect would have an impact only if it is applied on a large scale with an accumulation of significant savings. Otherwise, it will play a more educational role in society than being a real tool for accumulating resources.

The conditions of the effectiveness principle would not be met because of the lack of real representative large-scale data in support of the positive effects of the PRCAR.

The proportionality principle would require clear data on the lump sum of benefits so as to assess whether the risk-benefit ratio of the new policy is favourable. However, such data are currently not available in the Bulgarian context.

The necessity principle states that the proposed intervention should produce an outcome that cannot be achieved by another intervention that does not create such moral infringement. Healthcare reform is still ongoing in Bulgaria, with mixed results. The issue of scarce healthcare resources is undoubtedly a hot issue in the public health agenda, but jumping at the concept of patient responsibility before being able to safeguard equal access to health care and real health insurance coverage is rather extreme. Instead of freeing up more healthcare resources such an approach might exacerbate health inequalities since the population groups that are most likely to fall under the scope of the personal responsibility, are those that generally have lower health literacy and are of lower social class [35].

The least infringement principle would require that we first try other milder approaches to motivate people towards healthier lifestyle rather than cutting their access to healthcare procedures in case of unhealthy lifestyle.

The transparency principle requires justification of the new policy in public debate and as discussed above, such public debate is absent in Bulgaria for now.

The most difficult interpretation in our study would be the principle of reciprocity. In general, it means the obligation of society to mitigate the burdens imposed by public health regulations and actions by supporting an individual who is complying with public health mandates. How can society support an individual who is deprived of access to healthcare procedures based on his or her individual responsibility? This could mean the transfer of care from the health system to the social system. But is this really a resource-saving strategy?

### Limitations

The limitations of our study are related to the specificity and novelty of the investigated concept. We have covered the main documents regulating the Bulgarian healthcare system but we do not claim to have been able to encompass the multitude of existing legal documents of lower hierarchy. Also, our normative research could not be extended to the real healthcare practice, as this requires empirical research instruments.

## Conclusions

Although in a less categorical form and not directly related to access to specific planned treatments, PRCAR is embodied in the Bulgarian legislation. However, we couldn’t find enough supporting arguments either through the science of evidence translation from research to law or through the principles of public health ethics. There is no research supporting the introduction of PRCAR or proving its effectiveness to date. Thus, it is necessary to accumulate data on the applicability of these norms. Especially useful in this regard could be mixed method studies to combine quantitative data on a national scale with qualitative studies on the views of patients, users of social services, healthcare professionals, and the general public. Last but not least, wider public debate should be initiated both in the direction of clarification of the concept of PRCAR and the arguments for its eventual implementation in healthcare delivery.

## Data Availability

No datasets were generated or analysed during the current study.

## Abbreviations

PRCAR: personal responsibility of the patient as a criterion for allocation of resources
WMA: World Medical Association
